# On the History of Quinine, the Preparations of That Article, and Their Use in Medicine

**Published:** 1850-12

**Authors:** Theodore S. Bell

**Affiliations:** Louisville, Ky.


### WESTERN JOURNAL

OF

### MEDICINE AND SURGERY.

DECEMBER, 1850.

Art. I.—*On the History of Quinine; the preparations of that article, and their use in the Treatment of Diseases.* By Theodore S. Bell, M.D., of Louisville, Ky.

Dr. Paris remarks that "it is a humiliating thought to the medical profession that it is indebted for two of its most powerful remedies, to a quack and a savage," alluding to the introduction of mercury, as a medicine, by Paracelsus, and of cinchona bark, by a Peruvian. But the statements of Humboldt justify the belief that the discovery of the remedial powers of Peruvian bark claims a higher origin than that ascribed to it by Dr. Paris, and the most reasonable belief is that the Jesuits of Peru discovered the virtues of the cinchona barks. To that enterprising and investigating religious body all parties admit that Europe is indebted for the introduction of the great remedy, and their title seems clear from the fact that for many years, the cinchona barks went by the name of Jesuit powders, or were called after the names of some of the prominent members of Loyola's society. The discovery of the power of cinchona barks, by the Jesuits, is rendered highly probable from the peculiar characteristics of that body. That they spared no pains to cultivate every method of winning influence can scarcely admit of any doubt, and the practice of medicine, accordingly, was well adapted to their plans. Combined with the vast machinery of the ecclesiastical polity of the Jesuits, the dabbling in medical practice offered too rich a harvest to be neglected, and it was not neglected. It harmonised with their love of science, their fondness for philosophical pursuits, their botanical tastes, their self-sacrificing and earnest devotion to the sick, which led them to face pestilence with a courage that knew no faltering. They were generally men of science; they possessed the most cultivated minds of the age of which we speak. They spared neither labor nor anything else for the attainment of knowledge, and with wonderful diligence and success they investigated the resources of every new country, and investigations into Botanical subjects were among the most active of their pursuits. They knew infinitely more of Paraguay, in a short time after they entered that country, than was known by the natives, and their whole history, their active zeal for the advancement of the order; their devotion to science, and their love of medical practice, combined with the testimony of Humboldt seem to point to the Jesuits as the discoverers of the powers of the cinchona barks.

The introduction of Cinchona as a medicine marks an era in the healing art, and it met the fate that has often been meted out to great and valuable discoveries. Notwithstanding its well known virtues in South America, the first importations of cinchona bark into Europe, were unused for several years. Intermittent fevers and their kindred types of disease prevailed extensively, and were managed by the uncertain and unsuccessful remedies of the times, while a sure means of relief was lying as waste bark in the stores of the Apothecaries. Through the zeal and intelligence of Cardinal Lugo the remedy was at length brought into use, but soon fell into neglect, from which it was restored by an Englishman named Talbot, who used it as a secret remedy, for the cure of fever and ague. Its virtues were so marked and decisive that Louis XIV, of France, purchased the secret of Talbot, and made it public.* It is probable that Talbot discovered a better mode of preparing the bark for administration, than was known to the first crude methods of its administration. His mode must have differed from those in common use, or he could not have made a secret of his remedy. But, be this as it may, from the time of Louis XIV, to the present, the preparations of Cinchona have retained their place in the materia medica, and with unvarying good fortune.

I propose now to give a detail of the present position of some of the preparations of Cinchona bark, in the materia medica, and to show the important influence of these preparations, over various diseased conditions.—No members of the materia medica have commanded a more universal and undeviating confidence, and none are entitled to higher consideration. The salts of Peruvian bark are almost invariably successful in a vast range of disease, for which we know of no other reliable remedy,

---

* Talbot cured the Dauphin, son of Louis XIV, and thus gave notoriety to the remedy. See Eléments d' Histoire Naturelle Medicale. Deuxième partie. Botanique, 1849. Art. Rubiacèes. In the admirable memoir contained in that work, the physician who is curious about such information will find an ample supply of rich material. I have followed this authority in the spelling of Talbot's name—Pereira spells it Talbor.

and they are almost as essential as auxillaries in various maladies, for which they are not the main remedy. Whoever may take a comprehensive view of the almost interminable variety of malarious disease, which assumes a form of periodicity, and places a proper appreciation upon the remedial powers of the salts of Peruvian bark in the treatment of those affections, can be at no loss in giving these salts their true position among the agents that cure the most distressing maladies.

The ignorant and thoughtless often sneer at practical medicine as the science of guessing, and pronounce it non-progressive. Both assertions are devoid of even the semblance of truth, as may be easily proved by the history of intermittent and remittent fever. We can as certainly prognosticate the speedy recovery of a patient from either form of fever, under the administration of quinine, as a mathematician can demonstrate any of his problems; and the prognostication is founded as much upon reason and fact, as the demonstration is. There is quite as much guess work in the one case as in the other. Under the correct administration of quinine, the phenomena of those fevers move with the regularity of clock-work. And in answer to the statement that medicine is a non-progressive science, we may triumphantly point to the fact that forms of periodic disease that were formerly the opprobia of the profession, diseases that lingered through weeks and months, and left distressing traces of their existence upon all the after-life of the patient, are now not only curable, but are relieved in a few days, where they formerly required months to perfect a cure. Members of the profession, who are not very old yet in their vocation, can remember when weeks were considered the proper period for the course of a remittent fever, but it is no unusual occurrence now to see such cases cured in two or three days. Indeed, I rarely see our ordinary remittent fevers resist the genial influences of quinine when it has suitable auxillaries combined with it, adapted to the peculiar case.

The immense range, on this continent, of those forms of disease that originate in malaria, will aid us in placing a proper estimate upon that remedy which plays an all-important part in the treatment of the various maladies referred to. Some approach towards this estimate may be made, by any one who will trace on a map of the North American Continent, the following boundaries of autumnal fever, very graphically described in Professor Drake's work on "the principal diseases of the interior Valley of North America." In that work it is said of autumnal fever, a term which includes all varieties of intermittent and remittent fevers, "Being an endemic of all hot climates, we need not look to the shores of the Gulf of Mexico for a southern limit to our autumnal fever. Its base is, in fact, within the tropics; and prevailing, of course, in Havana and Vera Cruz, it is found wherever there are inhabitants, on the northern coast of the Gulf, between those two cities. In ascending all the rivers which discharge their waters into the northern arc of that closed sea, from Cape Florida round to the Panuco river, it is still met with; and sometimes, as we shall hereafter see, from the influence of local causes, displays greater prevalence and malignity than at shores farther south and on a lower level."

"In every other direction than the south, this endemic has its geographical limits. To the east its barrier is the Appalachian mountains, into the very gorges of which, however, it ascends by the valleys which penetrate their flanks. But as that chain is not found south of the thirty-third degree of latitude, it has, below that parallel, no eastern limit but the Atlantic ocean. To the southwest the Cordilleras of Mexico, and the southern Rocky Mountains, constitute the boundaries; while in higher latitudes, it ceases on the great plains of our western desert, long before we reach those mountains. From what can be collected out of the travels and expeditions of Lewis and Clark, Pike, Long, Catlin, Fremont, and Gregg, not less than from fur traders and Santa Fe merchants, it is almost unknown at the distance of three hundred miles from the western boundary of the States of Missouri and Iowa, and above the latitude of thirty-seven degrees north. To the north it does not prevail as an epidemic beyond the forty-fourth parallel, and ceases to occur even sporadically about the forty-seventh."

Within the immense area contained in the geographical limits thus described, may be found almost the entire series of maladies that spring from malaria, from the mildest grade of intermittent fever, through the multiform varieties of dysentery, cholera morbus, neuralgic affections, periodical toothache, earache, headache, intermittent fever complicated with cutaneous eruption, up to that formidable remittent fever, called yellow fever. And when we look at the immense area, described by Dr. Drake as the home of autumnal fever, when we gather from the census returns, what a multitude of human beings are congregated within that area, a large portion of whom are liable to the influence of the noxious agent of which we have spoken, the importance of the beneficent gift which disarms the pestilential effects of their evil power, rises at once to our view, and should admonish all that too much cannot be known of the proper uses and value of quinine.

The writer of these remarks was taught in the lecture room, that the use of quinine was altogether unnecessary in the management of intermittent and remittent fevers. The doctrine seemed so clearly demonstrated, that it was as implicitly relied upon as if it had been written by Moses on Mount Sinai. No one, after a long experience with Quinine or Fowler's solution, ever gave those remedies with more entire confidence in a favorable result, than I gave pills of Calomel, Aloes, and Rhubarb with a certainty of purging off the periodical return of the cold stage. It was a matter of amazement that agues could be so remarkably stubborn, as I found them, but then I had been taught that they were often very stubborn and intractable under any treatment. And even when necessity forced a surrender of the simple and beautiful theory to the stubborn requirements of experience and observation, the theory was comforted, and the patient was victimised, with an error scarcely less pernicious than that which was abandoned—that error was, that there was an absolute necessity of preparing the system for the use of quinine. I had been taught that there was a viscus in the human economy, called the liver, which played such an active, omnipresent part in all diseases, that there seemed to be no room for anything else in the human body. Its boundaries were very imperfectly understood. In a very large class of diseases of the brain, the liver was there; in the most of thoracic diseases, the liver was in the thorax; in nearly all abdominal diseases, the ramifications of the liver were to be distinctly traced. The brain, the lungs, the heart, pancreas, stomach, intestines, kidneys, uterus, bladder, etc., were mere satellites moving around a central sun, and that sun was the liver. In examining Dr. Stokes' daguerreotype of the Hepatic school of medicine, "in which the existence of almost every organ, except the liver, seems to be forgotten, and in which the creed seems to be that there is but one viscus, the liver, one source of disease, biliary derangement, and one cure, mercury;" it struck me, that I knew a man, whether in the body I shall not tell, or whether out of the body I shall not tell, who was covered by the picture as perfectly as any other sleeper ever was covered by a blanket. There seemed to be a necessity for looking up the other viscera, and for ascertaining whether they had any actual uses, and what were their conditions in health and disease. This was a work of some labor, but the rewards have been very great, and the improvement has been so useful, that the exercises that produced it, may be reasonably commended to others as conducive to the making up of the practitioner of medicine. The discovery of one additional viscus, where the existence of only one had been recognised before, is something in the way of progress; the full recognition of the movements and relations of all the viscera, in health and disease, is worth all the labor and perseverance that are necessary to its attainment.

It is somewhat singular with what tenacity an error frequently holds its ground in the practice of medicine. The necessity of putting the stomach and bowels in a special condition for the use of quinine, in the treatment of intermittent disease, and of remittent fever, is one that had such a feeble footstalk and is so palpable, that it is wonderful it could have lived long under the light of reason. The theory that gave the error much of its *animus*, was sufficient of itself, to overthrow it. The first link in the chain of evils was said to be weakened action of the heart; this brought on congestion of the vena cava; this produced derangement of the liver, and that opened Pandora's box. And yet we were taught to permit this state of things to go on day after day; to remove congestion at night, and have it renewed the following day, or the day after, as the case might be, thus introducing into practical medicine, the labors of Penelope! It is evident that each returning chill, each exacerbation of fever, must renew those derangements which physicians were endeavoring to remove, and that the proper mode of breaking up the concatenation of morbid phenomena would be found in that plan that promptly arrested the chill or the exacerbation of fever. And when the inquiry was made, what is there in the condition of the stomach and bowels, at the outset of periodical fever, that forbids the prompt use of quinine for its immediate arrest, it was impossible to give any satisfactory reason for delay. But custom was too strong, and the prejudices of early education are almost almighty. I broke through the meshes of early teaching with great difficulty, but got out with rapidity after the first attempt was made. A case of malignant chill occurred in this city, many years since, which created some noise at the time. I heard of the case with surprise, and upon inquiry, I was told that there was nothing unusual in the two chills that preceded the fatal one; that there was nothing whatever that induced a suspicion that the third chill would assume a malignant form. This aroused in me a determination never to permit a patient of mine to have a third chill, and this determination was strengthened soon after by the distressing case of a professional friend in the county of Jefferson. I had seen him but a few days before his illness, in full and vigorous health, apparently, and was surprised by a summons to visit him one evening, at his place in the country. I found him in the tenth hour of a malignant chill, presenting all the phenomena of a collapsed case of cholera. There was not a symptom of that stage of cholera absent from this case of malignant chill. Upon inquiry, I found that the victim in this case had paid little or no attention to the first and second chills, but had attended to his professional duties during the intermissions, and had trusted to an emetic, on the morning of the third chill, for arresting the ague fit. The emetic failed, and reaction never came on.

The two cases of malignant chill thus brought before my observation effected a thorough change in my views of the correct treatment of intermittent fever, and I have had no reason to regret the change. The rule adopted then, and persevered in ever since, was that the presence of intermittent fever was the exact preparation that required the use of quinine, and that the earliest administration that could be made of it, was the very best thing that could be done for the patient. Auxiliaries have sometimes been resorted to in this practice, but they were always subservient to the quinine—that is the chief and reliable remedy. In commencing this practice, which I was then persuaded was new, my intention was to amend the condition of the chylopoetic system after the intermittent malady was broken up, but in a long series of years, and in a great multitude of cases of all the various forms of intermittent and remittent fever known to this locality, I have rarely found it necessary to do anything after the ague was destroyed. The questions to be decided are: is it safe to administer quinine in intermittent and remittent fever, without preceding its use with emetics or purgatives? Is the patient likely to do as well under the immediate administration of Quinine, as under the use of it, after emetics or purgatives? Experience and widely extended observation answer in the affirmative, and go yet farther, for they testify that under the immediate exhibition of quinine patients are not only in as safe a condition as under the preparatory treatment, but that they are infinitely safer. They escape prolonged sufferings, avoid dangerous lesions, and are free from the risk of the malignant chill. These are abundant reasons for preferring the prompt use of quinine in periodical disease, but the advantages are much more numerous than those I have named. But I am anticipating the subject, and must return to the history of Cinchona barks, and their preparations.

In examining the early efforts of Chemists to ascertain the qualities of Peruvian bark, we have been pleased and surprised with the labors of Armand Seguin, an accomplished chemist, in the early days of this century, who had been the companion and colleague of Lavoisier. M.

Seguin commenced his investigations in 1802, for the purpose of determining the characteristics of true and false cinchona barks. He deserves a great deal of credit for his labors, and it is surprising that he should have come as nigh, as he did, to the discovery of the salts and alkaloids of cinchona bark, without stumbling on the discovery. He pursued his investigations after the characteristics of genuine Peruvian bark with a great deal of zeal, and with no little ability. Even Pelletier, in answer to a reclamation made by Seguin, compliments the labors of these analyses, and refers to the judicious remark of M. Seguin, that the quinquinas recognised as efficacious in medicine, are precipitated by the nut gall, while they are not precipitated by gelatin nor sulphate of iron.*

M. Seguin read three memoirs on the subject of his labors, to the Institute. In the first, entitled, "*Memoire sur un nouveau Fébrifuge*," read on the 28th of December, 1802, he undertakes to determine certain characteristics of the febrifuge principle in Peruvian bark, and contends that he has discovered those identical qualities in another article, and he declares that "the adoption of the new febrifuge is interesting to our commerce, which exports every year, more than three millions for procuring a principle which exists under our hand; to humanity in general, and in particular to that class worthy of the solicitude of this society, who are subject to fevers, by reason of the miserable character of their nourishment, and cannot procure, but with difficulty, on account of their indigence, the costly remedy which assures a cure." If the worthy analogist had reserved the denouement of his mystery for the end of his paper, one would have been tempted, while reading his marvellous accounts of

---

* Annales de Chimie et de Physique, vol. 16.

its powers, in various fevers, the exactness of the dose suitable for various ages, and the entire flourish of French rhetoric, to believe that M. Seguin had anticipated the French Academy in its wants, and prepared the discovery that was to displace Quinine, by identity of action. The inconveniences of the administration of Bark are clearly set forth, and the superior eligibility of his new febrifuge is duly proclaimed. When the old man set up a claim, in 1821, that he was the discoverer of the principle in Peruvian bark, that was then giving renown to Pelletier and Caventou, Pelletier killed him off, by a reference to the new febrifuge. This new febrifuge, which was to displace the best cinchona barks, which was said to destroy both continued and quotidian fevers, and when administered under the minute and prudent directions of M. Seguin, was said by him to remove the fever entirely in the third, fourth, or fifth paroxysm at most, was nothing more nor less, than animal gelatin! It is amusing to read the grave particularity with which he measured out the doses of this new febrifuge. For a sucking infant, about the age of one year or under, he ventured the hazardous experiment of giving from 24 to 65 grs. of gelatin! He runs through the various ages, up to 12 years, and for youth of that age he recommends from 2 gros to 12, and for all ages above 12, Seguin says we may employ from 2 gros to 3 ounces of the gelatin! He is careful about the periods of administration, and gives the contra-indications against its use with great minuteness. In summing up these, M. Seguin says, with an enviable naivete, "for, unhappily, the gelatin is not a universal remedy."

I have dwelt upon this singular memoir because it shows how a man of great capacity in his own line, may be on the verge of a great discovery, and by suffering himself to wander into matters of which he is profoundly ignorant, may not only miss the discovery, but become a driveller. Chemistry has too often left the laboratory, where it was at home, to dabble in practical medicine, to which it was an utter stranger. While M. Seguin confined himself to chemical analysis, he showed himself to be a chemist, but when he undertook the management of fever by chemistry, he sank into a dinner-pot. The records of medicine are filled with examples almost as notable as the one we have recorded of M. Seguin. When Pelletier and Caventou made their great discovery, they were content to place its results in the hands of the faculty of medicine, and left them to manage disease with it.

It is due to M. Seguin to say that two memoirs on cinchona barks, read to the Institute, subsequently to the one described above, were much more creditable to him. In them he confined himself more especially to chemical investigations. One of them was read on the 22d December, 1803, the other on the 19th March, 1804. In these he investigates the properties of the true cinchona barks, and points out the means for distinguishing them from the false barks, with considerable ability. Had it not been for his drivelling memoir on the new febrifuge, his name would have ranked much higher than it does. I repeat that M. Pelletier killed off his claim, in the 16th volume of the Annales de Chimie, as the discoverer of the Salts of Quinine, by a sarcastic reference to the gelatin. In reply to M. Seguin, he says* "en effet, dans un troisème memoire égálement lu à l'Institut, M. Seguin, partant de ses premières données, et trouvant dans la gélatine animale les caractères qu'il avait indiqués pour le principe fébrifuge du quinquina, n'hésite pas à presenter la *gélatine* comme très-propre à remplacer cette écorce." This response of Pelletier

* Annals de Chimie et de Physique, vol. 16, page 223.

seems to have extinguished all the aspirations of Seguin for being considered the discoverer of the salts and alkaloids of cinchona bark, for he never returned to the charge. He must have been deeply mortified to find how near he was, occasionally, to the great discovery, in some of his processes, and how completely he had missed it.

The memoir of Pelletier and Caventou, entitled "*Recherches Chimiques sur les Quinquinas*," presented to the Academy of Sciences, September 11th, 1820, is an admirable production, and is well worth the study of physicians, for it contains matter of vital interest to the profession. One of the most beautiful traits of this memoir is found in the manly and honorable reference made to the labors of preceding investigations. Full justice is odne to the merits of Fourcroy, Vauquelin, Reuss, of Moscow, and Gomès, of Lisbon. The acknowledgment of the services of Gomès is full. That chemist preceded Pelletier and Caventou in the discovery of the organic salifiable base in the quinquinas, which was of great use to the discoverers of the sulphate of quinine. It would extend these remarks too greatly, were I to undertake to show the important bearings, on chemical science, of the discovery of cinchonine, by Gomès. Pelletier makes it a part of a suite of discoveries, which "marked an epoch in the science."

The memoir of Pelletier and Caventou contained a full account of their treatment of cinchonine, sulphate of cinchonine, hydrochlorate of cinchonine, nitrate of cinchonine, phosphate of cinchonine, arseniate of cinchonine, acetate of cinchonine, oxalate of cinchonine, tartrate of cinchonine, gallate of cinchonine, and an analysis of the grey bark of cinchonine. It would be difficult for the physician to point to any other labors of the chemist's laboratory that have had as important an effect upon practical medicine as the material contained in the admirable memoir, which I have described. The thanks of the medical profession, and of countless multitudes of the human family are due to Pelletier and Caventou for the beautiful and bountiful gift bestowed on humanity, by the discovery of the salts and alkaloids of Peruvian bark. The discovery has had a great deal to do with the progress of practical medicine; it has greatly abridged human suffering, and has saved a vast multitude of lives. It has rendered successful, easy, and reliable, the treatment of diseases that were formerly intractable, uncertain in their issues, and often fatal. The Peruvian bark, in powder, is an exceedingly nauseous and revolting dose; so much so that a patient would be justified in debating whether it is best to have a chill or to take the medicine. I have tried both, and should decidedly prefer the chill to swallowing Peruvian bark. If the Peruvian bark were now, when the whole civilized earth is almost in daily communication, and "day to day uttereth speech, and night to night showeth knowledge," as universally used as quinine is, we should constantly hear of its uncertain results, of long, tedious, annoying attacks of intermittent fever; of dreadful sequela, belonging to repeated engorgements of the viscera, all of which would be laid at the door of the remedies. We have but an imperfect picture of the state of things that existed in medical practice, when anti-periodic medicines were confined to bark in powder, decoction, tincture, etc., but imperfect as the picture is, enough is known to suppress a desire for a return of such a state of things as then existed. Pelletier and Caventou in discovering the sulphate of quinine, discovered almost a new continent for practical medicine, and they should be regarded as benefactors of the medical profession, and through it, of the human race.

Notwithstanding the perfectness of the practical part of the discovery of Pelletier and Caventou, chemists were by no means agreed as to the truth of all the conclusions of these eminent masters in science. In 1827, MM. Henry and Plisson undertook to settle the questions that were agitating the minds of chemists on the subject of the discoveries of Pelletier and Caventou. Their memoir, published in the 35th volume of Annales de Chimie, is exceedingly interesting, but its details belong rather to the Pharmaceutist, than to practical medicine. Columbus discovered the new world under a false theory, but the world forgets the theory in the practical result—the same is true of the labors of MM. Pelletier and Caventou. Even if they erred, they conferred a great practical fact upon the world, which throws into shadow any errors that may have accompanied its advent. That, which Dr. Bartlett truly and beautifully says of the works of Louis, may be said of the discoveries of Pelletier and Caventou:—"like new planets added to a solar system, they have quietly but irresistibly wheeled into their orbits, from which they are henceforth no more to be jostled, than the planets are from theirs."

The most eminent medical observers bear unequivocal testimony to the reliable properties of quinine. Chomel and Double, (it would be well for practical medicine if they were types of a larger class than they are,) say "that the salts of quinine and cinchonine, particularly the sulphate, have nearly constantly succeeded in those cases, where those medicines may be looked to as means of relief." I am sure there are few physicians who have used the sulphate of quinine, with a well regulated judgment, who will hesitate in corroborating the judgment of MM. Chomel and Double. The use of that article in disease; the time and mode of giving it; the object for which it is given, and the circumstances that forbid its administration, are a study in themselves which will amply reward any one who sets about it in the proper way, and uses due diligence and application in the pursuit. Let no one victimize himself and others, with the vain imagination that all is known, that should be, of the uses, qualities, and powers of the Sulphate of Quinine.

In entering upon the uses of quinine, I owe it to myself to say, that I am not attempting to write anything new on the subject. The design of this paper is to gather all the rays of light on the administration and benefits of sulphate of quinine, that can be brought within the compass of the essay. After what has been said here, it is scarcely necessary for me to declare that I look upon that medicine as one of the most valuable that has arisen among the tortuous paths of practical medicine. And most cordially do I subscribe to the opinions of Professor Bartlett, on the position of quinine in practical medicine. Of the anti-periodic qualities of cinchona and its preparations, he says: "there is no substitute for these. They are universally relied upon for this purpose. In all countries, and at all periods, since the discovery of the properties of this incomparable and invaluable substance; amidst all the conflicting dogmas of different medical doctrines, Peruvian bark has never failed to sustain its reputation, and to answer the expectations that have rested upon it. Amidst the manifold uncertainties of medical science, and the perpetual contingencies of medical art; amidst the disheartening scientific infidelity which has lately been taking possession of the medical mind, shaking to its foundations the firm old faith in the potency of drugs, and threatening to overturn and demolish it altogether,—it is gratifying and consolatory to feel and to know, that here at least we stand upon solid ground, that here we may hold,—that there is at least one great and important therapeutical relationship definitively and positively ascertained and established, defying alike the open assaults of quackery from without, and the treacherous machinations of indolent skepticism from within." If, under all the numerous disadvantages that attend the administration of the barks of cinchona, the classic and philosophic Senac was justified in calling Peruvian bark, *the divine remedy*, what may we not be justified in saying of the sulphate of quinine?

I think that the discoveries of Fourcroy, Vauquelin, and Gomès, made prior to the great success of Pelletier and Caventou, were not in a greater degree superior to the knowledge before their time, than the sulphate of quinine is superior to all the other preparations that are made from quinine and cinchonine. Of its prëeminence I feel no kind of doubt, and this conviction rests on the slow and cautious accumulations of experience and observation. For eighteen years I have been in the habit of making almost daily prescriptions, either of preparations of Peruvian bark, or of the salts or alkaloids made from that article, and all my experience and observation go to the establishment of the fact that the sulphate of quinine is not only the most reliable of all the salts of cinchona, the most eligible for administration, and most invariably successful, but it stands prëeminent over every other means that I have seen used. I have seen the most pernicious intermittents yield as readily to its administration, as initial inflammation of the brain does to the lancet and cold water. I have often been called upon to treat dropsies, enlargement and induration of the liver and spleen, and serious affections of the stomach and bowels that had followed the cure of intermittent fever by cinchona bark, or thoroughwort, or some of the very many quack remedies that abound in the west, and I have seen all these morbid actions subside rapidly, day by day, under the influence of the sulphate of quinine. I wish it were possible for me to say of any other article of the materia medica, what I can say of quinine, and that is, that under a use of it for eighteen years, it has never disappointed me but once in intermittent fever. It has been used as the main remedy, generally, and as the only one often, in the treatment of intermittent and remittent fevers, and its good effects have uniformily presented themselves. Its control over the relapses of those forms of fever is not the least certain and useful of its powers. But a detail of the uses of the beneficent gift which chemistry has bestowed upon practical medicine, will be given under appropriate heads.

In a former part of this essay, I have spoken of the pernicious heresy that is inculcated in the doctrine of the utility and necessity of preparing a patient for the use of the sulphate of quinine. What possible reason can be urged for the existence of such a doctrine, I cannot imagine. Indeed, I have never heard any urged, except the mere declaration of the opinion, but it is usually declared as oracularly as a professor of mathematics would announce any one of Euclid's axioms. Of the fatal tendencies of the heresy, I have already presented proofs, arising from the results of delay, in arresting the periodical recurrence of dangerous congestions. In a recent number of the British American Medical Journal, a highly instructive case, on this point, is given by Dr. Mack, of St. Catherines, West Canada. The patient in this case, "had been living in a shanty on the margin of a stagnant pond, near the debouchment of the Welland Canal into Lake Ontario." He was seized with a tertian intermittent, for some weeks, and employed for its cure, a nostrum called "cholagogue." It was supposed that the cure was effected, because the paroxysms were interrupted, but doubt and great uncertainty hang over all these methods of cure, no matter what appearances may be. A physician would have discovered in the complaints of the patient that he was not relieved of his disease. He, however, went to his work, for a few days, and while making some iron bands, took a moderate draught of cold water. He was suddenly seized with severe pain, and Dr. Mack found him writhing in great agony, referring the seat of pain to the left side of the chest and abdomen. The group of symptoms showed that the sufferer was in the most imminent danger, and, after suffering sixteen hours, he died. A *post-mortem* examination revealed the fact that the spleen was ruptured, and I think the morbid appearances justified the following conclusions of Dr. Mack: "in this case it is probable that a rupture of the splenic vessels, occurred during the congestion accompanying the cold stage of ague, first gave rise to an extravasation of blood within the splenic membrane. (This might have been increased at each subsequent congestion.) The afflux of blood following the reception of the cold water into the stomach at the time of the attack, ruptured the disturbed capsule and peritoneum, and a fatal effusion resulted." It is unnecessary to dwell at length upon the fatal lesions that may result from delay in breaking up the proximate cause of dangerous congestions. They are too familiar to all who have practiced in a malarious region. These dangers may not always result in perilous lesions, but even the morbid effects stand prominent enough, as beacons, to admonish a prompt removal of the periodical attack. Mere functional derangement, as a consequence of persistent intermittent fever, in many cases makes itself felt in all of the subsequent life of the patient. Truth, experience, observation, fidelity to the profession of medicine, and a faithful guardianship of the welfare of the sufferer, all imperatively require of the physician the utmost promptitude in arresting the chills. When I can cut the first paroxysms off in the middle of its efforts, I always do it, and never permit a second one to show itself, when I am called in time to interfere. As long as we are ignorant whether the attack of chills may terminate in the malignant variety; of what fatal lesions may occur; of what functional disorders may be left as heir-looms, by child's play with the disease, that length of time will physicians be called upon to use promptly the resources of their art in breaking up that state of things that is so frequently perilous, and the existence of which is always unnecessary. I have seen patients purged day after day for weeks, the physician apparently waiting on providence in the hope that something would turn up to justify the use of quinine. And when the state of the patient at length compelled the use of that medicine, the patient's system was not as well "prepared" for the use of the quinine, as it was before the scientific preparatory treatment was commenced, yet to their amazement the patient recovered without any evil effects from the quinine. I should think that such facts would have an effect upon the after-practice of the physician, but there are men who will walk through mud-holes in a well-beaten path, especially if the path has been beaten by themselves, rather than turn out of the road. Upon such the lights of experience shed their rays in vain; with them all the phenomena of disease, and recovery, are as accidental as the throws of the dice, and they forget that in practical medicine, fortunate numbers may turn up, if the dice are properly loaded. The man who does not improve under the teachings of experience is a pitiable object, but the physician who does not advance in such a school is baleful.

In 1838 I was taught that it is not absolutely necessary to wait for the subsidence of fever, in order to commence the use of quinine. Mrs. H. P., wife of the editor of the Louisville Daily Journal, was attacked by a formidable chill; reaction came on slowly, and the fever continued up to the commencement of the second chill. The reaction from this was slower than after the first; and the symptoms were of such a grave character that I

felt well assured that a third chill would be fatal.—The old notions of preparatory treatment, and the necessity of the subsidence of fever, for the use of quinine, rose in all their authority before me. I found it difficult to shake off this incubus, but feeling well convinced that the patient would die without the quinine, I ventured on the conclusion that she would do no worse with it. I fully explained to the patient the real dangers of her case, and told her of all the perils that might reasonably be looked for under the administration of the quinine during the exacerbation. I felt very solemn, and talked in funereal tones, and wonder that I did not send for a priest, in order to have the last offices of religion convenient. The patient preferred the least evil, and resolved to take the quinine. It was given in doses of two grains, every two hours, and I called every two hours to look up the direful results of the treatment. Greatly to my surprise the case seemed to be doing well, but previous training would not permit me to believe even the evidences of my own senses, until my patient reached the period for the third paroxysm in a most comfortable state of convalescence. I cooled down then, and got within the range of reason, and began to think that the fashionable doctrines in the use of Quinine in intermittent fever, needed some reform.

There is a volume of practical matter in the following pithy sentence, in Maillot's *Traité des Fièvres:* "That treatment which, in a malarious region, attempts to remove local irritatious before administering the sulphate of quinine, which waits to convert a grave into a simple intermittent, before resorting to febrifuges, prepares for itself inevitable reverses." As far as extent of experience, remarkably fine powers for observing, a cool, cautious, discriminating judgment, and a philosophic cast of mind can confer authority upon the opinions of a man, I know of none who can boast of more of these elements than Maillot. And I assure the reader of these remarks that if his practice lies in a malarious district, he will find the principle contained in the above sentence from Maillot invaluable in practice, alike conducive to his own reputation and the welfare of his patients. Such a reader should remember that in recent cases of intermittent and remittent fever, he cannot, easily, too soon commence the use of quinine; and in chronic cases, that article should be a leading element in the treatment.

Within the last few years a great change has taken place in the mode of administering quinine, especially, in intermittent fever. The practice formerly was to commence in the apyrexia and give two grains of quinine every 2 hours until the return of the period for the recurrence of the paroxysm. This plan was supposed to be the only safe one; a great fear was felt of the stimulating effects of the medicine, and it was quite common "to watch the effects," as the phrase goes. Although many physicians stepped out of this well worn rut, the experience of the physicians of the United States army, in Florida, first gave prominence to the great superiority of administering quinine in a large and decided dose, at once. The paper, read on this subject before the National Institute, first taught the value of the practice, and it has steadily increased in the estimation of observant physicians, from that time to the present. Where an interval of from fourteen to eighteen hours can be found, a single dose of twenty grains of sulphate of quinine, administered to an adult, at the commencement of the apyrexia, and from four to six grains to a child, are the best means of managing the disease. This plan is quite as certain in its result, in arresting the paroxysm, as the divided portions of quinine were; the disturbance of the patient, the breaking of his sleep, and the consequent injury to the nervous system, are all avoided by the decided dose. A patient can take twenty grains of quinine, and rest secure against any further return of the chill.

I have not found that females, except sojourners from the South, bear the large dose of quinine as well as men do, and I have generally preferred giving them a ten grain dose, and repeating it in five or six hours.

These remarks apply especially to recent cases; in chronic forms of the disease, other measures are also necessary. In these cases the liver, stomach and bowels demand some attention, and mercurial cathartics should be combined with the quinine treatment. While it was formerly permitted for dangerous congestions to take place, through fear of quinine, it is not a little singular that that medicine was regarded as the best agent for the removal of those congestions.

Intermittent fever sometimes commences with intense pain in the head, and in many of the joints. The febrile symptoms are formidable, thirst insatiable, and the general aspect of the case alarms the friends of the sufferer. In this state of things a free bleeding, from a large orifice, with the application of cloths, that have been steeped in hot water, to the head, not only gives prompt relief to the patient, but secures several additional hours of apyrexia, for the greater control of quinine over the next paroxysm.

In 1835 I had an attack of intermittent fever, and became the subject of frequent relapses. They became so annoying that I consulted a number of medical friends, whose age and experience, I hoped, might furnish me some means to avoid the troublesome returns of the disease. They assured me that they knew of no medical means that would answer the purpose. It seemed to me that I was condemned to a life of suffering and inaction, and the subject occupied my thoughts almost constantly. At length I struck out an idea that ought to have been palpable at first. It is, that the quinine that interrupts the action of the cause of the paroxysm, if continued long enough, will remove the cause altogether, and I acted upon the thought at once in my own case. From that time to the present I have had no relapse, and as fifteen years have elapsed, I think there is little danger of one now, and that the principle has fairly tried itself. In taking quinine for this object, it is necessary to use it but once a day, in doses varying from three to ten grains. Under this course of treatment I have seen but little difficulty with relapses. Some years after this use of quinine, I was gratified in finding that Dr. Graves, of Dublin, was advocating the principle, which had been successful in my own case.

In medical books on intermittent fever, recommendations are often made as to what is to be done, when quinine fails. I cannot understand the meaning of this, because I have never seen but one case in which quinine failed. In that case, all the anti-periodics that I had ever heard of, failed quite as signally as the quinine. I am persuaded that scarcely one case in a thousand will fail to be cured by the sulphate of quinine, and I cannot see any good reason for seeking a remedy to take the place of one so uniformly successful.

In simple remittent fever I have generally pursued the course described in the treatment of intermittent fever. The great object is to make as perfect a remission as possible, and this can usually be done by cold ablutions of the body and spine; cloths with hot water in them, to the head, and a judicious use of the lancet. These means, combined with the administration of quinine, are all that I usually find necessary in the treatment of simple remittent fever, and the success has not only been gratifying, but I have been able greatly to facilitate the cure of such cases, in comparison with the former treatment. It is seldom that the lancet, purgatives or nauseants have to be used. The quinine may be used in the early periods of remittent fever, in the same way that it is given in the intermittent form. Of course, after serious inroads are made upon the healthy functions of the viscera, other means than the quinine must be resorted to, but even then, that medicine is an important adjunct.

In the early stages of congestive fever, the sulphate of quinine is the all-important remedy. That disease is a pernicious intermittent, and must be promptly mastered, or the patient will be in extreme peril. I have seen the intermissions disappear entirely after the second paroxysm, and when that is the case, it is seldom that the physician prescribes for a graver series of symptoms. I was called upon, some years ago to visit a gentleman in consultation, in a county adjoining Jefferson county. He was laboring under a severe form of congestive fever, all tendency to intermission or remission had disappeared. The treatment had been what is called "the active kind"—the patient had been liberally bled, purged and blistered, and when I visited him he was severely ptyalised. Under all these series of depletion, the patient had continually grown worse. The suggestion was made to the attending physician that all depletory measures should be abandoned, because it was evident that as far as they had been carried, they had produced no amelioration in the system; that it was true, it might be urged that those measures had not yet been carried far enough, but in my judgment, if they were carried any farther, they would end in relieving the patient of his evil symptoms and life at the same time. The suggestion of sulphate of quinine as the remedy was promptly opposed, but the use of it was finally agreed upon, on condition that I would take the responsibility. I did so; the quinine was at once commenced, and given in liberal quantities. From the beginning of its use the patient began to improve, and his amendment was so rapid, that in a few days, I retired from the case. I give this as a specimen of the difference between the depletory treatment of congestive fever, and the quinine treatment. I could multiply details of similar cases to a great extent, but it is unnecessary to burthen the reader with any thing more than the practical indications. I am not writing an essay on intermittent, remittent or congestive fever, but am merely endeavoring to show the value of the early use of quinine in these diseases.

The neighborhood of Louisville occasionally shows cases of congestive fever that would rival the most formidable description of that disease given by Morton or Torti. In a paper on cholera, I quoted Senac's description of the pernicious or congestive fever, and asked then, what modern writer has given a more perfect description of cholera than is found in Senac's description of congestive chill or fever? There is not a symptom of the one that is not to be found in the other. Senac says: "at another time the skin is suffused with a deep red, which is lighter, however, in some parts than in others. But more frequently a kind of *sudor Anglicanus* breaks out. In many cases this flows abundantly from the forehead and breast; it is everywhere warm, and the more plentifully it flows, the more intense is the fever. After a certain space of time, sometimes longer, sometimes shorter, the sweat becomes cold; the pulse is depressed and in some cases creeping. Under these circumstances, some patients experience a tightness and oppression about the præcordia, others a sense of heat and burning in the stomach, while all are affected with restlessness, tossing, and a suppression of urine. The appearance of the body itself bespeaks impending danger, for the flesh becomes of a livid or bluish color." In speaking of cramps or spasms, Senac says: "these spasms are communicated to the external parts and produce in them various motions and effects. The same symptoms therefore occur in intermitting fever which take place in cholera morbus. There exists, however, this difference, that in fever these symptoms end when the paroxysm ends: whereas in cholera there is no respite," etc. It would be no difficult task to make out a fair description of cholera, from Senac's detail of the phenomena of malignant chill. It is not a little singular that Senac compares the symptoms to those of cholera. The phenomena of these two formidable diseases being so nearly alike, shows the necessity of prompt and energetic treatment. If the remedial measures can be resorted to in an early stage of the disease, the relief may be prompt and effectual, but I know of no disease in which delays are more dangerous than in congestive fever. Time is so precious, that it is almost everything. I have always been satisfied that the nervous system is the main sufferer in the commencement of congestive fever, and that the sanguineous derangement is a sequence of the morbid condition of the functions of the nerves. Hence the great value of a mustard sinapism to the spine, in the forming stages of congestive fever. I have seen a sinapism over that region, with thirty and forty grain doses of quinine, every one or two hours, break up the most formidable attacks of congestive fever. But if the case has become complicated with sanguineous derangement, mercurials must be combined with the quinine. If there is great restlessness, an opiate should be given, and for this purpose I prefer the liquid muriate of opium, prepared after the following formula, of the Boston Medical Journal:

Pulv. gum opii,
Acidi hydrochloric ää ℥i,
Aqua distill. ℥xij,
Spiritus gallici ℥iv,
Make by displacement.

Of this from twenty to thirty drops may be given. Its chief advantage is that it does not distress the head. It must never be forgotten that congestive fever is a formidable disease, and that it requires prompt, judicious, and energetic treatment.

Quinine has been strongly recommended in the treatment of what is called Asiatic cholera, and from what I have seen of its effects, I regard it as an important remedy in that disease. Dr. C. W. Bell, of India, looks upon cholera as a species of ague, and warmly recommends the use of quinine in its treatment. If we may credit his report of cases, quinine must be regarded as a valuable remedy. Dr. Bell's mode of giving it is the following:

℞. Quinæ. disulph. grs. xij to ℈i,

Ferri sulph. grs. ix,

Acid sulph. dilut. M. xl,

Aqua puræ, Oiss, repeated from quarter of an hour to every hour, according to circumstances.

I think that the analogy between pernicious ague and cholera is very palpable. In reading Morton and Torti on malignant chills, it is difficult to feel that they are not describing the Great Disease of our era. But I wish that the analogy between the two diseases were more perfect in one important point. In malignant chills at the outset, there is a tendency to some reaction, no matter how feeble that may be, it is something that gives a little vantage ground for the treatment. In that state of things that is generally recognised as cholera, the downward tendency is uninterrupted when left to nature, and this constitutes a material difference between the two diseases. Analogical reasoning is always dangerous; there is in it a species of Virgil's "facilis descensus Averni," that should admonish of the difficulties of getting back on open ground. Medical men should, of all others, be guarded in the reception of all doctrines that rest on analogical proof, for that has been the bane of the profession too long. The strict, rigid, and undeviating plan of finding pure facts, and then restricting the inference within the legitimate powers of the fact, may be a very slow process, but it is a sure one, in the attainment of knowledge.

That malignant chill and cholera proceed from the same remote cause, is, I think, as reasonable an inference as can be drawn from facts. We know of no two causes that can so nearly produce identical effects, unless the causes are as nearly alike as the effects are. The characteristics, the season of prevalence, the localities at which the two diseases appear, the similarity of result in hygienic measures for the prevention of both, are a portion of the elements of identity, enough, at any rate to justify us in resorting to what may be found the best remedial measures for one disease, as the best for the other. That which will remove the *Sudor Anglicanus,* the violent cramps, the severe vomiting, the shrunken features, suppression of urine, watery purging, and blueness of skin, in a case of malignant chill, should be equally successful in removing the same symptoms in a case of cholera. This is safe and tenable ground.

The treatment I have detailed as successful in congestive fever, I have found of great value in cholera. There is a state of things in congestive fever in which that disease is curable, and there is a stage in which all remedies are unavailing, and this description is equally applicable to cholera.

In order to corroborate the value of the suggestions of Dr. C. W. Bell, already quoted, I submit the following cases, furnished me by Dr. Charles L. Lyle, of this city. I have frequently seen the same admirable results from the use of quinine in similar cases, but prefer the reports of Dr. Lyle to any of my own. Dr. Lyle reports as follows:

—— Garrett, aged about 40 years, by occupation a stone-mason, was employed on the island near the falls, getting out stone, during the fall and winter of 1848. He was very much exposed in his occupation, working in the water a great deal. He lived in a small board shanty on the island, and frequently slept in his wet clothes. On the evening of the 24th Dec., 1848, I was called to see him. His first exclamation, when I entered the room, was that he had cholera; that he had been purging and that he felt as he did when he had cholera in 1832. His appearance was that of a man with a congestive chill. The countenance was anxious, breathing labored, extremities cold and shriveled; he looked blue, as if his blood was not sufficiently arterialised. The pulse was scarcely perceptible at the wrist; his legs and arms were occasionally cramped. The indications of cure were at first to relieve the circulation; and to restore the warmth of the body. In order to fulfil these indications, I had him rubbed with hot turpentine and Granville's Lotion along the spine; sinapisms were applied, as soon as they could be prepared, to the extremities, and nearly all over the body. I had injections of sugar of lead and laudanum administered, to arrest the purging. I gave brandy, tinct. capsicum, camphor, tinct. assafetida, laudanum, calomel and opium, and probably other stimulants, all of which seemed merely to answer the purpose of holding the disease in check. I then commenced giving x grains and ℈j doses of quinine every half hour, with manifest improvement, which increased as I increased the quantity of quinine. In a short time my patient was relieved. He relapsed twice, but was relieved each time by the use of quinine, calomel, and opium.

Case II.—Willis Woolfolk, aged 17 years, had been exposed on the river during the spring of 1849. Sometime in May, (I forget the date,) I was called to him.

He had been purging and vomiting water, that was almost colorless. He made no complaint; said that he was not sick, but felt very weak; that he had been purging occasionally during the night, and the discharges were of the same character I have mentioned above. He said they were not attended with pain. The expression of his countenance was care-worn, skin cool, pulse quick and feeble. With some difficulty I succeeded in getting him to take medicine to relieve this purging and vomiting, which I succeeded in arresting in a short time. He was approaching collapse. I gave him brandy and other stimuli, which he rejected as soon as they were swallowed. He complained of great thirst. I had him covered with hot turpentine, and applied mustard and hot bottles and bricks to his feet and legs. I then applied a large tumbler, exhausted of air, as a cup over the epigastrium, and gave x grain doses of quinine every half hour with marked benefit. The patient was permitted to swallow small portions of ice to allay his thirst; after a time this improvement seemed to cease. I then gave, after a short interval between them, two doses of quinine, ʒss each. Perfect reaction soon took place, and he speedily recovered. I soon succeeded in procuring proper discharges from his bowels in this case. The object of the tumbler, was to compel the patient to retain the quinine, it being almost impossible for the stomach to reject anything while under the influence of an exhausted tumbler, inverted over the stomach.

Case III.—A negro man named Charles, aged 28 years, was employed in a rope-walk, on Washington street, near Brooke; you understand its locality, and have often noticed the filth that is washed there from almost every direction. It is low and flat, and there is an annual deposit from high water. When I saw the boy, he was vomiting and purging rice-water; extremities cold and shriveled; pulseless at the wrist, (he had suffered with diarrhea for 48 hours); countenance anxious; voice so husky that you could not understand him; great thirst; discharges involuntary. In accordance with directions that I had left for the hands in case of an emergency, he had received active, and what I considered good treatment. Injections of lead and laudanum, small and frequently repeated doses of calomel, lead, and opium; he had been cupped to arrest vomiting, rubbed with hot turpentine; mustard plasters had been applied; in fact he had received quite energetic treatment. In addition to the above, he had drank near a quart of brandy. I continued such of these means as I could, and gave quinine ℈j every fifteen minutes. Finally, believing my case almost hopeless, I dissolved ʒss doses of quinine in as much brandy as he would take, and gave it every few minutes, and injected him with the same. In a short time he went to sleep, and slept as if he were narcotised, for several hours. His pulse became very slow, there seemed to be almost a minute between each inspiration, which was deep and full, but not stertorous. The interval was so long, that I was afraid that he would never breathe again. From this sleep he awoke relieved. The only ill effect of the quinine was a slight roaring of the ears, which subsided in a few days. I may mention that the patient had taken opiates enough to kill an ordinary case, before taking the quinine.

I have used quinine, very much in the manner I have mentioned above, in quite a number of cases of cholera, in the treatment of that stage approaching collapse. I cannot say that in a single instance I have not been pleased with it. I have never used quinine alone, but combined with such other means, as occurred to me, would be of service in the treatment of the case. I have used quinine in every stage of cholera with decided benefit, and always, for its full effect, increase the dose until the object is attained.

The usefulness of the tumbler, applied as described by Dr. Lyle, for controlling irritability of the stomach, cannot be too highly estimated. I have never seen it fail to prevent vomiting, and have heard patients plead for its removal, in order to give them a chance to puke. A very respectable practitioner of medicine in this city, who devoted himself most assiduously in attendance upon the victims of cholera, in what is known here as the plague spot, in the lower end of the city, was himself violently attacked with cholera. When I saw him his efforts at vomiting were almost incessant, and they were among the most painful I have ever seen. From the moment of the application of the tumblers, as cups, over the epigastric region, all vomiting ceased, and the patient declared that from the moment of the application of the tumblers, until they were removed, he felt as if he had no stomach, and said that he did not believe that any emetic could have acted upon him while the tumblers remained on him. I have used them in the way recommended, a multitude of times, and their success has been unvarying. Dr. A. P. Elston used them extensively, for the same objects mentioned here, and he bears testimony to their uniform efficiency. He mentioned to me a case, in which he used the tumblers and arrested the vomiting; upon their removal, it returned, and it was again arrested by the re-application, and these alternations continued until the patient was relieved of the disease.

The value of this remedy for vomiting is the greater from its eligibility. It may generally be applied in a few minutes. It is only necessary to pour into a tumbler a small quantity of brandy or alcohol, and shake the liquid so as to smear the inside surface of the tumbler with the alcohol. Then throw into it a lighted piece of paper, and while the alcohol is burning, invert the tumbler promptly over the epigastric region. The vacuum formed by the burning alcohol, gives the external atmosphere an opportunity of making a considerable pressure, and the tumbler remains fixed, frequently, for hours. If the vomiting were one of the dangerous symptoms of cholera, we should have a great control over the disease, by this simple remedy. But unfortunately, the gastric movements are more distressing and annoying, than dangerous, and patients often rapidly sink into death after the complete arrest of the vomiting and purging, and many fatal cases occur without any vomiting.

I feel persuaded that quinine is a more important remedy for cholera, than it has credit for being, and that it deserves a judicious trial of its powers. No one pretends that it is a specific; but it will be found an important adjuvant to other remedies. Its use, for success, needs a cool and discriminating judgment. While its powers over intermittent fever are almost universally acknowledged, there are forms of that disease that will not bear it. A few years since an endemic intermittent fever showed itself in China, so complicated with intestinal irritation, that the quinine aggravated the disease, and it was necessary to relieve the intestinal disturbance, before the quinine could exert its powers. But in cases of this kind, we may obtain the benefit of the quinine, by combining with it calomel, and the aqueous extract of opium, or what is perhaps better, the valerianate of morphia, a new preparation, made by Professor Horsford, for Dr. M. Wyman, of Boston. I have found the combination of the aqueous extract of opium, with quinine, of great service, in the intermittents and remittents of children, when there is much irritatability of the stomach.

*Rheumatism.*—Quinine has been highly commended in the treatment of rheumatism, and there can be no doubt of its value in some forms of that affection. But there are many cases of the disease, and probably many varieties of it, over which quinine exerts no kind of influence. It certainly exhibits no more power in rheumatism than is obtained from nitrate of potash or iodide of potassium. The physician who undertakes the treatment of any particular disease, with one specified remedy, used alike under all the varying aspects of morbid action, is doomed to much disappointment. He must recognise the fact that there is more than one agent in the Materia Medica, and must make suitable combinations with his favorite medicine, in order to suit the varying phases of disease, as they show themselves under the modifying influences of age, sex, temperament, habits of life, &c.

M. Vinet urges the use of quinine as a remedy for acute rheumatism. I have seen great benefit from its use, and I have seen it fail, where I had strong reason for believing it would succeed. M. Vinet says that "the cases in which it proves most efficacious, are those in which the general and local symptoms are best marked. In cases in which it procures a prompt cure, it may prevent the cardiac complication, and in those in whom it is slower in its operation, it does not seem to favor the occurrence of this, which, however, when it does occur, requires the usual appropriate means for its removal. The beneficial effects are generally the more promptly produced as the dose is large, such being often observed after a moderate amount of perturbation of the nervous system." The dose may rise from 15 to 45 or 50 grains in the twenty-four hours. And whenever the cinchonism is severe, I have generally found it promptly relieved by mustard sinapisms to the ancles and wrists.

*Neuralgia.*—The remarks I have made on the treatment of rheumatism with quinine, apply with equal force to the treatment of neuralgic diseases. These affections spring from too many causes, to be obedient to any routine treatment. Where they are not caused by inflammation of the nervous sheath, or by ulceration, or mechanical pressure, quinine, in doses of from 10 to 20

grains, given every 3 or 4 hours, usually relieves the disease in a short time. I have seen a number of singular and very severe neuralgic diseases here this fall, and in all of them, the quinine exerted a controlling power, and relieved all the patients. Last spring I saw an unusually severe case, which was complicated with another disease. After the relief of the complication, the neuralgia, located in the hip-joint, was unusually distressing. The slightest movement on the part of the patient produced the most poignant agony. For many days and nights he sat in a chair, with his feet resting in another chair. I used the vin. tinct. colchicum, and the iodide of potash, with great freedom, and then tried the sulphate of morphia, but without mitigating the sufferings of the patient. He could neither sleep nor eat, and when he was nearly worn out by suffering, vigilance, and starvation, I commenced the use of the sulphate of quinine with him. It was given in 10 grain doses every 2 hours, and the third dose relieved the pain so much that the patient was able to go to bed and in a short time he fell into a refreshing sleep. The quinine was continued about a week. This fall the patient had a renewal of the attack, and it was promptly relieved by the quinine. I have seen the same marked benefit from the quinine in a multitude of cases, but I have also seen it fail, without being able to ascertain any good reason for the failure.

*Intermittent Affections of the Eye, Ear, &c.*—All who have practiced in malarious regions are familiar with these forms of disease. They are very regular in their periods, though they are often obscure. I have seen a depletory treatment followed in these cases, for a length of time, greatly to the injury of the patient, and without any relief of the distressing pain. If the intermitting character of the disease is recognised, there will be no difficulty in giving relief. I have rarely seen any of these forms of periodic disease resist quinine twenty-four hours.

When the disease is located in the eye, it is at times very deceptive. It resembles ophthalmia so much, that it is sometimes difficult to tell which of the two diseases is to be prescribed for. But if the intermissions are watched, a clue is easily obtained for the treatment. These anomalous forms of periodic disease usually appear about the superciliary ridge, in the eye-ball, or some of the appendages, in the ears, and in the teeth. I have known leeches applied to the brow, or the eye-lids, an onion or garlic poultice to the ear, and under the entreaties of the agonised patient, a tooth is sometimes drawn. It is obvious that these erroneous forms of treatment are bound to fail. The quinine, however, acts like a charm, and the diseases soon disappear under its management. In a malarious district, it is safe to watch all forms of anomalous *malaise*, for intermittent action. And it is well enough to remember that a place may almost lose its malarious character, for years, and then resume it again in an extended form. The records of medicine, especially among the Germans, contain many notable examples of this truth. It is in this intermittance of decided intermittent and remittent fever, that we may reasonably look for those anomalous kinds of malarious disease that are often so obscured as to deceive any one who is not experienced in the diagnosis of such diseases. Among these anomalous forms is one in which a man may be easily betrayed into error, by the very means from which he would hope for the greatest certainty. The disease to which I allude is *intermittent asthma*, in which the stethescope might lure a man from the path of truth. I have a great deal of confidence in Laennec's discovery, but it is not a perfect substitute for all other sources of investigation. There are some other things that demand attention, as well as auscultation and stethescopy, especially when those schools are crowded with pretenders. I hold that it is useful, at least sometimes, for the physician to remember that there are causes of disease, that demand quite as much consideration as the physical signs. The physician who would base a treatment of intermittent asthma upon the physical signs might commit a blunder that would not benefit his patient. While remembering modern explorers and observers, let us not forget that such men as Hippocrates and Galen have lived, and that all the wisdom of modern schools may learn something valuable from them. Our treatment of intermittent asthma must be based upon a knowledge of malaria, and quinine is the remedy, of whose constant success any one may assure himself, who may have an opportunity of giving it a fair trial.

In that most distressing symptom, so often met with in malarious districts, and often intractable — *an obstinate hiccup*, M. Mendiere says, he has found quinine the most successful remedy. It has often succeeded after the failure of all other remedies. Dr. Johnson advises the combination of some preparation of opium with it, and when this is deemed useful, I should prefer the muriated preparation referred to in a former part of this paper.

There are few persons in a malarious region who are not familiar with that *engorgement of the spleen*, usually denominated ague-cake. The testimony to the value of quinine in this affection is quite uniform, both in Europe and America. M. Chomel has used it with great success. M. Bally pointed out the engorgement of the spleen as the cause of relapses in intermittent fever. His reasoning seems to be quite conclusive, and if true, we accomplish two important results in the use of quinine; we remove the engorgement of the spleen, and break up the tendency to relapse. The views of M. Bally have been amply confirmed by M. Nonat. The latter says, that the quantity of quinine required to effect a permanent cure of an ague, and of an induced enlargement of the spleen, is almost always in a direct ratio with the amount or extent of this enlargement. He adds: "after a great many experiments, I have satisfied myself that the dose of quinine should vary from 12 to 40 or 50 grains in the twenty-four hours, and that it must be proportioned to the increase in the size of the spleen." The medicine should be continued until that viscus is restored to its natural size, and this may sometimes be hastened by combining the bromide or iodide of potassium with the quinine. When the stomach will not bear the large doses of quinine that are requisite, it is strongly recommended to use it in enemata, of course in larger quantities than when it is taken into the stomach.

*Yellow Fever.*—I have no personal experience in the treatment of this disease, but it would scarcely be proper to pass by the triumphant success of the southern profession in the treatment of yellow fever, with the sulphate of quinine. The treatment is called the "abortive method by quinine." This treatment should be commenced within twenty-four or thirty-six hours of the attack. The plan is to resort to a hot mustard footbath, and a purgative enemata—from 20 to 30 grains of quinine, with 25 or 30 drops of laudanum, or one or two grains of opium, or the fourth of a grain of the sulphate of morphine are given at one dose. Great success is reported, under this treatment, and we have no reason to doubt the truth of the reports. Dr. Fenner speaks from personal observation, and is entitled to the respect and confidence of his professional brethren. But it must be remembered that time is all-important in order to secure the success of the use of quinine. While the derangement is *entirely functional*, the abortive treatment is successful, but when visceral engorgements occur, we must resort to other means. The same principle holds good in intermittent and common remittent fever. In ordinary agues, when the period of attack anticipates its time, we know that the patient is growing worse; the anticipation is due to changes of visceral action. When the attack is postponed beyond its usual time, it is an evidence that the functions of the viscera are improving, and we know the patient is recovering. Some of the French observers, in Paris, have sounded notes of alarm over the large doses of sulphate of quinine, given by M. Brecquet, but I think needlessly. There is no poisonous property in quinine, and when we wish to overwhelm a disease at once, we must give large doses of the medicine. I have often taken twenty grains at a dose, and in its administration in that dose, I have seen no evil effects, save in women. They do not bear the large doses as well as men do. In the "Experimental researches on the action of quinine, especially in large doses," by M. Brecquet, the following facts were noted: "the sulphate in doses exceeding three grains is absorbed in from half an hour to two hours, and produces its physiological effects in another hour. These will continue for about half an hour. A dose of fifteen grains in six hours, continues to manifest its influence for from five to six hours. Thirty grains administered in two hours, produces symptoms lasting from twelve to fifteen hours. When the sulphate has been administered for several days, the effects continue many days after it has been withdrawn. The medicine is completely eliminated at the end of ten or twelve hours after small doses, and in about forty-eight or seventy-two hours after large doses.

"Women and children are more susceptible of its influence than men; and the stature and strength of the individual modifies its effects. Loss of blood increases also its influence, diminishing its stimulating and increasing its depressing action. Opiates act in a similar manner, while alcoholic stimlants have a reverse operation."

M. Brecquet ascertained that the sulphate is the most active of all the preparations of quinine; the alkaloid and cinchonine are identical with the sulphate in their action, but the cinchonine is one-third less powerful, and quinoidine equals the sulphate in its action on the nervous system, but is much more irritating to the alimentary canal. The sulphate is most active in solution. In enemata it is more active than when given by the mouth, but the effects are of shorter duration. M. Brecquet has no faith in quinine frictions, ointments, lotions, and other endermic methods, and I fully agree with him.

Southern and western physicians are fully advised of the value of quinine in certain stages of *Pneumonia.* The good results are well marked. The combination of tartrate of antimony and potass, with quinine, is often invaluable in this disease, and in all forms of intermittent and remittent fevers, where these affections are complicated with thoracic disturbance.

One of the most valuable qualities of quinine is the facility with which it assimilates with other medicines in complicated disease. If any given case demands tartar emetic, or calomel, or opiates, or digitaline, or other means, while we obtain the full benefit of the adjuvant, the power of the quinine is not diminished nor interfered with.

A great deal of verbiage has been uttered as to the peculiar properties of quinine. One contends that it is a stimulant, another regards it as a sedative, another as a tonic. These manifestations are equivalent to a controversy as to whether opium is a narcotic, an astringent, a stimulant, or a sedative. Quinine displays all the qualities ascribed to it by the contending sects, and its action depends upon the state of the viscera, and the mode of administration. The cultivators of medical art should rejoice to know that sulphate of quinine has a variety of powers of which judicious use may be made in the treatment of disease.

*Substitutes for Sulphate of Quinine.*—A former number of this Journal contained a translation of an offer of a prize, by the French Society of Pharmacy, for the discovery of a substitute for quinine that should possess all its good qualities, and another prize for the discovery of a method of making quinine, from its elementary constituents. It is evident, from the terms used in the programme of the society, that they do not place much faith in the reported virtues of the articles that have been vaunted as being equal to the sulphate of quinine, and I think physicians generally coincide in the skepticism. The society made its offer in full view of all that is alleged of the anti-periodic powers of Salicene, Bebeerine, Adansonia Digitata, Phloridzine, Hydrocyanoferrate of Quinine, the amorphous quinine, commended by Liebig, and a host of other articles. An extended experiment is now going on in the United States' army in order to ascertain the value of salicene, but if the results of private practice are of any force in determining such questions, it is not in the least degree probable that salicene will displace quinine. I have made an extensive trial of the salicene, and its services have not impressed me favorably. Nor can I speak any more highly of any of the other proposed substitutes for the sulphate of quinine. Such of them as make any approach to quinine in one feature are sure to be defective in some other important element. And it may serve to take the keen edge from expectation, founded upon the offer of the French Society of Pharmacy, of a prize for the manufacture of quinine from its elemental constituents, to look into chemistry to see how far hope may be justified. In chemistry, bodies consisting of the same elements, united in the same ratio, are called isomeric bodies, and there is a notable instance that illustrates the fallacy of expecting too much from chemistry's "counterfeit presentments" of nature's handy work. Bebeerine, one of the proposed substitutes for quinine, is isomeric with morphia, but the action of the two articles is widely variant. Messrs. Maclagan and Tilley very properly say: "that similarity of physiological properties does not depend upon similarity in the properties of their constituents. It seems probable that the mode in which their atoms are grouped has an important share in modifying their physiological actions."

I do not wish to be understood as placing too low an estimate upon the qualities of the articles named as substitutes for the sulphate of quinine. I have but little faith in their general powers, when brought into comparison with quinine. A new article equal to that sal would be an inestimable boon, but I am slow to believe that it has yet been attained, or that it is likely to be. It is not sufficient that a medicine shall be equal to the sulphate of quinine as a febrifuge; among other good properties, it must show the same beneficent combining powers, with various other medicines, that are found to belong to quinine in an eminent degree.

I must here close these remarks upon the medical properties of quinine. Of the various combinations of medicines with quinine, I have not spoken, because ample information on those points is easily accessible to the physician. My chief desire has been to record the present position of quinine, in practical medicine.

Of the beneficent boon conferred on practical medicine by the labors and science of Pelletier and Caventou, it would be difficult to speak too highly. In a single point, this gift of chemical science is invaluable, if in no other. I allude to the difficulty of adulterating sulphate of quinine, and the ease with which its impurities are detected.

Even as far back, as the latter part of the past century, avarice and villainy had attained such perfection in adulterating the cinchona barks, that all the leading chemists of the age had their attention strongly directed to the means of testing the true barks. But the world of intermittent and remittent fever was a prey to designing knaves, and unscrupulous swindlers, and the counters at which the counterfeits could be detected, were far distant from those who most needed the service. What the geometric lathe has done for bank-note engravings, the discovery of Pelletier and Caventou has done for practical medicine, and for suffering humanity. The adulterations of quinine though frequent, are easily detected. But the adulterations of Peruvian bark are easily made, and are detected with difficulty, except by the practical chemist. The general dealer is rarely able to bring the Peruvian barks to the test. And even under the present limited demand for Peruvian bark, in the hands of the druggist, great adulterations are made; what they would be if the world were as dependent upon that article, as it once was, may possibly be conjectured somewhere within the range of probability, by a man of very fertile imagination.

*Adulterations of Quinine.*—The necessity of a pure sulphate of quinine for the treatment of disease is so great, that I think it important to present the most recent methods for detecting impurities in that invaluable medicine. "Pure quinine may be obtained in long acicular crystals, but it is generally in delicate fibrous and soft crystals of a pearly aspect, and flexible like amianthus; it is efflorescent, intensely bitter, and requires about 740 parts of cold, and 30 parts of boiling water for solution. It is soluble in 80 parts of alcohol, (sp. grav. 0.850), and much more soluble in boiling alcohol. The salt readily fuses, looking at first like melted wax; it then reddens, and begins to be decomposed; in the air it burns, producing at first a bulky charcoal, which by continued heat, is consumed without residue. It has the curious property of becoming luminous when heated to about 212°; its phosphorescence is increased by friction, and electricity is, at the same time manifest."*

The usual adulterations are water, sugar, gum, starch, ammoniacal salts, and earthy salts, such as sulphate of lime, sulphate of magnesia, and acetate of lime, salicene, and sulphate of cinchona. When pure sulphate of quinine is heated to 212°, to deprive it of its water of chrystalization, it should lose only from 8 to 10 per cent. in weight. *Sugar* may be detected by dissolving the suspected salt in water, and adding precisely as much carbonate of potassa as will precipitate the quinine. The bitter taste of the quinine being removed the sugar can be tasted. The sugar can be separated from the sulphate of potassa, by evaporating gently to dryness, and then dissolving the sugar by boiling alcohol. *Gum* and *starch* are left, when the impure sulphate of quinine is digested, in strong alcohol. In the *Cours de Chimie Générale*, of Pelouze and Fremy, it is said that "we may discover the presence of an inorganic matter in the salt by burning a small quantity on a platina plate," [a thin strip of glass gradually heated will answer the purpose,] "the sulphate of quinine leaves no trace of residue, if it is pure." "*Ammoniacal salts* are discovered by the strong odor of ammonia given off when the sulphate is put in a warm solution of potassa. *Margaric acid* is sometimes mixed with sulphate of quinine; it is detected by its insolubility in dilute hydrochloric acid." The tests for sugar and salicene, according to Dr. Nevin, are as follows: to 2 grains of the suspected salt add 4 drops

---

*Brande's Manual of Chemistry, sixth edition.

of sulphuric acid and 8 of water; if fatty matter or starch be present they remain, if absent, the salt is dissolved, and on applying heat, sugar is indicated by *charring*, and salicene by *a red color.* The pure salt is not effected by this application of sulphuric acid." If Boracic acid is used to adulterate quinine, its presence may be detected, by dissolving the suspected salt in alcohol, and burning it. If Boracic acid is present, the alcohol burns with a green flame; if a few drops of sulphuric acid are added, the greenness is increased. The sulphate of cinchonine is sometimes used for adulterating the sulphate of quinine. Liebig says: "if the quinine is thus adulterated, the mixture is not soluble in 10 parts of hot water." For the detection of this impurity Pereira directs that a solution of the suspected salt in water, be precipitated by potassa; "collect the precipitate, and boil it in alcohol; the cinchona crystalizes as the liquor cools, while the quinine remains in the mother liquor." This is probably accurate enough, but M. Henry points out a more complete method. It is, however, complicated and troublesome. I think it important that the physician shall verify the purity of the sulphate of quinine he uses, and the methods here commended are easily used. The celebrity of a great name as the manufacturer of the medicine is only a guaranty that the article was pure when it left his establishment; there, unfortunately, the protection ceases. If physicians are careful in these matters, apothecaries will be vigilant; if the physician is negligent, the apothecary is apt to be careless.

*The incompatibilities of Sulphate of Quinine* are the alkalies and their carbonates; lime water; tartaric acid; the soluble tartrates; and all vegetable tinctures, infusions, and decoctions containing tannin.*

---

* Neligan's Medicines and their uses, &c.

I have thus reviewed at length the past and present position of the sulphate of quinine. I regret that the interruptions of practice have left me so small an amount of time for the preparation of this essay, that it is necessarily imperfect. The great importance of the remedy, and its invaluable powers in the hands of the intelligent physician, plead powerfully in favor of a thorough understanding of its capacity. If the gratitude of the world shall ever approach so near the region of the beautiful, the true, and the proper, as to raise monuments to those who have benefitted humanity by the healing art, the monument to Pelletier and Caventou will not be the least conspicuous of the collection.*

---

*Since this article was in type, the writer accidentally saw, for the first time, Professor Austin Flint's remarks on Quinine, published some years ago in the New York Medical Journal. It is a source of regret that the character of that publication was not known in time to render it a tribute of respect in this monograph, but the writer cannot withhold the expression of the gratification he feels in finding the views advocated here, sustained by one who deservedly stands as high in professional estimation as Professor Austin Flint, editor of the Buffalo Medical Journal. There can be but little doubt, that Professor Austin Flint was the pioneer in the practice of treating intermittent fever, with one decisive dose of the sulphate of quinine.

